# Outcomes of Cancer Surgery During the COVID-19 Pandemic: Preparedness to Practising Continuous Cancer Care

**DOI:** 10.1007/s13193-020-01250-z

**Published:** 2020-10-19

**Authors:** C. Ramachandra, Pavan Sugoor, Uday Karjol, Ravi Arjunan, Syed Altaf, Rajshekar Halkud, R. Krishnappa, Purushotham Chavan, K. T. Siddappa, Rathan Shetty, V. R. Pallavi, Praveen Rathod, K. Shobha, K. S. Sabitha

**Affiliations:** 1 Department of Surgical Oncology, Kidwai Memorial Institute of Oncology, Bengaluru, 560029 India; 2 Department of Head and Neck Oncology, Kidwai Memorial Institute of Oncology, Bengaluru, 560029 India; 3 Department of Gynec-oncology, Kidwai Memorial Institute of Oncology, Bengaluru, 560029 India; 4 Department of Oral Oncology, Kidwai Memorial Institute of Oncology, Bengaluru, 560029 India

**Keywords:** COVID-19, SARS-CoV-2, Pandemic, Cancer surgery, Cancer care, Surgical outcomes

## Abstract

The COVID-19 pandemic has placed unprecedented pressure on healthcare services. Deprioritisation of nonemergency clinical services and growing concerns of adverse outcomes of COVID-19 in cancer patients is having a deleterious impact across oncologic practice. We report cancer surgery outcomes taking into account the acuity of the COVID-19 situation. A prospectively maintained database of the Department of Surgical Oncology was analysed from 1st May to 30th June, 2020, to evaluate the perioperative outcomes, morbidity and mortality following major surgical procedures. A total of 359, preoperatively, tested negative for COVID-19 underwent surgery. Median age was 52 years with 26.7% (*n* = 96) above the age of 60 years. Sixty-one percent (*n* = 219) patients were American Society of Anaesthesiology grades II–III. As per surgical complexity grading, 36.8% (*n* = 132) cases were lower grades (I–III) and 63.2% (*n* = 227) were complex surgeries (IV–VI). 5.3% (*n* = 19) had ≥ grade III Clavien-Dindo complication, and the postoperative mortality rate was 0.27% (*n* = 1). Major complication rates in patients > 60 years were 9.3% in comparison to 4.1% in < 60 years (*p* = 0·63). The median hospital stay was 1–10 days across subspecialties. Postoperatively, repeat COVID 19 testing in 2 suspected patients were negative. Our study showed that after screening, triaging and prioritisation, asymptomatic cases may undergo cancer surgeries without increased morbidity during COVID-19 pandemic.

## Introduction

Coronavirus disease 2019 (COVID-19), an emerging and evolving situation, has ensued unprecedented pressure on hospitals and ICU’s necessitating rapid redeployment of staff and resources towards the management of COVID-19 cases with deprioritisation of nonCOVID-19-related nonemergency clinical services; concurrently, lockdown, public anxiety, disruption of primary care services and hospital referrals of symptomatic cases have caused significant collateral damage to cancer care [1, 2].

Modest delay in diagnosis and timely interventions in patients with cancer may result in tumour progression and may result in the likelihood of metastasis [3]. Recent safety concerns regarding aerosol generation from endoscopy, cystoscopy and surgery [4, 5] have further exacerbated cancer care.

Several cancer centres drastically deescalated their services after preliminary reports from China showed that Covid-19 outcomes are significantly worse among patients with cancer, hence outcome data following cancer surgery are sparsely reported from COVID hotspots around the world. We aimed to report outcomes of 359 cancer surgeries from a COVID hotspot geographical location.

## Materials and Methods

A prospective database of the department of surgical oncology was analysed from 1st May to 30th June, 2020.

### Hospital Preparedness, Patient-Directed Initiatives, Screening and Prioritization of Patients for Surgery

We were cognizant of the magnitude of the problem in neighbouring states and early ringfencing of substantial resources during the nationwide lockdowns of varying intensity from 25th March to 30th June 2020, helped to mitigate the coercion caused by COVID-19 pandemic.

We were quick to adopt best practices and guidelines (PRINT ISSN No. 2277-8179/doi: 10.36106/ijsr) for cancer treatment during the pandemic as prescribed by Indian Association of Surgical Oncology (IASO)COVID-19 guidelines [6] and the Tata Memorial Centre COVID-19 working group [7].

Good responders to neoadjuvant treatment, who were likely to benefit from potentially curative resections and patients with fewer comorbidities, were given the highest priority. Surgery was avoided for poor prognostic diseases and in whom interventions were expected to have marginal benefits.

A COVID-19 testing lab was set up in the campus, and a routine preoperative COVID 19 testing was performed in all patients from 1st May 2020. The test was performed on nasopharyngeal as well as the oropharyngeal swabs by real-time reverse transcriptase polymerase chain reaction (RT-PCR) method.

### Data Collection and Statistical Analysis

The demographic, perioperative and postoperative recovery data were obtained from the prospectively maintained database. All statistical analyses were carried out with the Statistical Package for the Social Sciences version 21 (SPSS Chicago, IL, USA). Continuous variables are described as median unless stated otherwise. Distribution of variables was identified by descriptive analysis. A 2-tailed independent proportion test (*Z* test) with 5% level of significance was used to calculate the difference between two proportions with respect to age group, American Society of Anaesthesiology (ASA), surgical complexity grading versus complications.

### Ethics

The data of the present study were collected in the course of common clinical practice, and, accordingly, the signed informed consent was obtained from each patient for any surgical and clinical procedures. The study protocol conforms to the ethical guidelines of the World Medical Association Declaration of Helsinki: Ethical Principles for Medical Research Involving Human Subjects adopted by the 18th WMA General Assembly, Helsinki, Finland, June 1964, as revised in Tokyo 2004. No approval of the institutional review committee was needed.

## Results

A total of 359 surgeries were performed between 1st May 2020, to 30th June 2020, of which343 (95.5%) cases were elective surgeries while 16 (4.5%) were planned emergency surgeries. Demographic and operative outcomes are illustrated in Table 1. Median age was 52 years with 26.7% (*n* = 96) above the age of 60 years. 61% (*n* = 219) patients were ASA grades II–III. As per surgical complexity grading, 36.8% (*n* = 132) cases were lower grades (I–III) and 63.2% (*n* = 227) were complex surgeries (IV–VI). Head and neck surgeries accounted for 27.5% (*n* = 99) cases, 37.7% (*n* = 37 cases) required pectoralis major myocutaneous pedicle flap for reconstruction of head and neck defects. Breast cancer surgeries constituted (18.1%, *n* = 65 cases) followed by gynec-oncology surgeries (14.4%, *n* = 52)) and GI cancer surgeries (13%, *n* = 47 cases). 5.3% (*n* = 19) had Clavien-Dindo (CD) ≥ grade III complication, and the postoperative mortality rate was 0.27% (*n* = 1). Perioperative outcomes across cancer sites are provided in Table 2.

**Table 1:** Patient demographics, surgical procedure and postoperative outcomes

Sl. no	Variables	*n* = 359 (%)
1	Age (years), median	52 (range 4–84)
2	Gender	
	Male	143 (39.8%)
	Female	216 (60.2%)
3	ASA grade	
	I	140 (39%)
	II	212 (59.1%)
	III	7 (1.9%)
4	Cancer site	
	a. Head and neck oncology	99 (27.5%)
	Composite resection + PMMC	37
	Wide excision ± ND	28
	Thyroidectomy	11
	Parotidectomy	5
	Maxillectomy	4
	Total laryngectomy	10
	Neck dissection	3
	Orbital exentration	1
	b. Breast oncology	65 (18.1%)
	Modified radical mastectomy	46
	Breast conservative surgery	15
	Lumpectomy	3
	Microdochectomy	1
	c. Gastrointestinal oncology	47 (13%)
	Subtotal gastrectomy	7
	Proximal gastrectomy	1
	Gastrojejunostomy	2
	Small bowel resection anastomosis	1
	Pylorus preserving pancreatico-duodenectomy	5
	Hemicolectomy	4
	Abdominal-perineal resection	3
	Low anterior resection	5
	Hartman’s reversal	1
	Posterior exenteration	1
	Diversion stoma	8
	Ileostomy reversal	9
	d. Thoracic oncology	11 (3.06%)
	Transhiatal esophagectomy	8
	Lobectomy	1
	Chest wall resection with LD reconstruction	2
	e. Genitourinary oncology	21 (5.8%)
	Radical nephrectomy	4
	Radical cystectomy with ileal conduit	2
	Total penectomy ± GND	4
	Partial penectomy ± GND	2
	Adrenalectomy	1
	Bilateral scrotal orchidectomy	3
	High inguinal orchidectomy	1
	GND	4
	f. Musculoskeletal oncology	12 (3.3%)
	Total knee replacement	2
	Arthrodesis	1
	Amputation	3
	Wide excision with NCS	1
	Wide excision with fibular grafting	1
	Internal hemi-pelvectomy	1
	Fibulectomy	2
	Hip disarticulation	1
	g. Sarcoma Wide excision	3 (0.8%)
	h. Gynec-oncology	52 (14.4%)
	TAH + BSO+ BPLND	12
	Primary cytoreductive surgery	24
	Interval cytoreductive surgery	11
	Vulvectomy + GND	2
	Type III radical hysterectomy	1
	Bilateral salphingo-ophorectomy	2
	i. Others	49 (13.6%)
	Biopsy	26
	DL Scopy + biopsy	4
	EUA + Cystoscopy	2
	Feeding jejunostomy	9
	Emergency trachesotomy	7
	Debridment	1
5	Grades of surgery	
	I	20 (5.5%)
	II	24 (6.7%)
	III	81 (22.6%)
	IV	141 (39.3%)
	V	56 (15.6%)
	VI	37 (10.3%)
6	Clavien-Dindo 30-day morbidity	
	Overall complication	105 (29.2%)
	Grade I–II complications	86 (23.9%)
	Grade III–IV complications	19 (5.3%)
	Mortality	1 (0.3%)
7	Reexploration	10 (2.8%)
8	Readmission	8 (2.2%)

*PMMC* Pectoralis major myocutaneous flap, *ND* neck dissection, *LD* Latismus dorsi, *GND* groin node dissection, *NCS* nail cement spacer, *TAH + BS0* total abdominal hysterectomy with bilateral salphingo-ophorectomy, *BPLND* bilateral pelvic lymph node dissection

**Table 2:** Peri-operative outcomes according to cancer site

Variables	Head and Neck	Breast oncology	GI oncology	Thoracic oncology	GU oncology	Skeletal oncology	Sarcomas	Gynec-oncology	Others
Grades of surgery									
I–III	03 (3%)	04 (6%)	20 (42.5%)	0 (0%)	07(33.3%)	05 (41.6%)	1 (33.3%)	2 (3.8%)	33 (67.3%)
IV–VI	96 (97%)	61 (94%)	27(57.5%)	11 (100%)	14(66.7%)	07(58.4%)	2 (66.7%)	50 (96.2%)	16 (32.7%)
Duration of surgery (median, Min)	210	90	210	240	150	140	90	180	20
Median blood loss (ml)	350	120	350	300	130	150	140	300	50
Median number of days in ICU	2	None	3	2	1	None	None	None	0
Postoperative stay median, days	9	1	7	10	7	5	6	5	1
Readmissions	3	0	2	0	1	0	0	0	2
Reexplorations	2	3	2	0	0	0	0	0	3
Grade III–IV complications	3	3	4	2	2	0	0	2	3
Mortality	0	0	1	0	0	0	0	0	0

Ninety-six patients were above 60 years age, of which 9.3% developed major complication in comparison to 4.1% in < 60 years (*p* = 0·63). Twelve percent of patients with ASA I developed ≥ grade III complications in comparison to 14% in ASA II–III (*p* = 0.06).

All the preoperative patients underwent testing for COVID 19. Postoperatively, two patients were suspected to have contracted COVID-19; as determined by clinical and chest X-ray findings, a repeat RT PCR–based COVID testing in these two patients were negative. None of the operated patient in the study period had a detrimental postoperative course.

## Discussion

The global pandemic of respiratory disease caused by the novel human coronavirus (SARS-CoV-2) has caused untold suffering, loss of life and upheaval in society. The pandemic has led to massive redirection of health care resources to treat the surge of COVID-19 patients, hence delivery of health care to the nonCOVID-related diseases including cancer patients has been significantly disrupted.

Patients with cancer might be immunocompromised by the effects of antineoplastic therapy, supportive medications such as steroids, and the immunosuppressive properties of cancer itself, additionally, patients with cancer are often older (i.e., aged ≥ 60 years) with one or more comorbidities, making them vulnerable to increased risk for COVID-19-related morbidity and mortality. Initial reports suggested that patients with a history of or active cancer might be at an increased risk of contracting the virus and developing COVID-19-related complications [8–10], yet generalising this to overall population of patients with cancer would result in deferring treatment.

Cancellations of cancer surgeries has resulted in pandemic driven protocol modifications in an attempt to bridge the intersection of COVID-19 and cancer treatment because suspending cancer treatment will have untoward consequences. An understanding of the disease course of COVID-19 and factors influencing clinical outcomes in patients with cancer is urgently needed for treating cancer patients effectively through turbulence.

Bengaluru was not the first city in India, to see a surge of COVID19 cases, but it has had the most rapid increase in case numbers and currently has the most cases overall in Karnataka State. Our city has been described accurately as the hotspot of the pandemic in the state. Currently, some other institutions are dealing with the same challenges we are facing, and many others undoubtedly will face these challenges in the weeks and months to come.

At Kidwai Memorial Institute of Oncology (Regional Cancer Centre at Bengaluru, India) despite having to deescalate operations by about one-third, the department of surgical oncology and allied specialities made the decision to continue graded response in providing cancer care based on, for a centre with an annual registration of more than 25,000 new patients even a slowdown in clinical services is likely to have a deleterious impact on outcomes and a high plausible that surges of population infection, lock downs, resource competition and diagnostic bottlenecks could recur over the next few years and augment the delay in oncological care and its consequences [11].

GLOBOCAN 2018 projected national cancer incidence of 1.1 million and 0.78 million cancer deaths with a case fatality rate of 22.9% [12]. It is pertinent to note that, in India, close to 1.3 million COVID-19 confirmed cases prevail (till date of submission of the manuscript) with 30,659 deaths, having a case fatality rate of 2.35% [13]. The projected national cancer incidence burden in 2020 will be 98.7 per 100,000 population (1,392,179 patients) as a conservative estimate [14]. It is important to be mindful that, with case fatality rate of about 22.9%, the cancer mortality in absence of definitive surgery will far exceed the mortality due to infection with COVID-19. These observations also compelled us to continue cancer surgeries during the pandemic.

Triaging, prioritisation of surgical cases and rationalization of services were key instrumental in continuing the surgeries uninterruptedly. During the initial phase of pandemic, low risk patients, i.e., young age, patients with fewer comorbidities and in whom potentially curative resection can be performed were given a priority.

The median age in the present series is 52 years. ASA I patients constituted 31%. There was no difference in 30 days CD complications when patients over 60 years were compared to those younger than 60 years of age and when ASA I was compared with ASA II–III. Our results reflect a cautious approach adopted initially that has gradually widened in scope with increasing confidence.

The low rates of major complications in our series reflect appropriate case selection and early adoption’s best surgical practices and stringent standard operating procedures. Our surgical outcome experience is in concordance as reported by Shrikhande et al. [15].

The applicability of our experience to the COVID positive cancer patients is yet to be established.

Administrational rationale of having an in campus COVID testing laboratory, early in the course of pandemic, strengthened case selection and segregation of nonCOVID-19 patients. Till date of submission of the manuscript 79,266 samples have been tested from various parts of Karnataka at in campus laboratory, total tested positive cases were 2992 of which 166 were of cancer patients, who are presently under quarantine.

As per clinical parameters and radiological findings, two patients were suspected to have contracted COVID-19 during the postoperative period; they were subsequently nursed in isolation, and repeat RT-PCR test was negative, and the morbidity was attributed to nonCOVID causes.

Laparoscopy can lead to aerosolisation of blood borne viruses [16–18]. Specific evidence to COVID-19 and the risk for aerosol transmission during laparoscopy are lacking; a conscious decision was taken to err on the side of safety, hence minimally invasive surgeries (laparoscopic and robotic services) are temporarily suspended until the operative rooms are equipped with a closed smoke evacuation/filtration system with ultra-low particulate air filtration (ULPA) capability.

## Conclusions

The surgical outcome measures of our study, i.e., ICU stay and major morbidity validates, continuing elective cancer surgeries. Our reproducible approach and results serve as a springboard for continuing cancer surgeries during the pandemic. Until an effective vaccine becomes available for widespread use, it is imperative that surgical oncologists remain focused on providing optimal care for cancer patients, while managing the demands that the COVID-19 pandemic will continue to impose on our health system.
